# Depth measurement of molecular permeation using inclined confocal microscopy

**DOI:** 10.1371/journal.pone.0214504

**Published:** 2019-03-27

**Authors:** Kenji Kikuchi, Shunsuke Shigeta, Takuji Ishikawa

**Affiliations:** 1 Department of Finemechanics, Graduate School of Engineering, Tohoku University, Aramaki, Aoba, Sendai, Japan; 2 Graduate School of Biomedical Engineering, Tohoku University, Aramaki, Aoba, Sendai, Japan; Pennsylvania State Hershey College of Medicine, UNITED STATES

## Abstract

We report a new technique for the high time-resolved depth measurement of molecular concentration distribution in a permeable hydrogel film with micro-depth precision. We developed an inclined observation technique in a laser-induced fluorescence (LIF) system, based on confocal microscopy, which measures the concentration distribution in the depth direction at less than micrometre intervals. The focal plane of confocal microscopy was tilted to enable simultaneous depth scanning in the microscopic field of view inside the permeable substrate. Our system achieved real-time and non-contact depth measurement of concentration distribution in the permeable hydrogel film. Simultaneous depth concentration measurement was realised with < 1 μm/pixel resolution over a maximum depth range of 570 μm, depending on the tilt angle of the stage and optical conditions. Our system measured the concentration of fluorescence materials based on the fluorescence intensities at several depth positions with a minimum concentration resolution of 1.3 nmol/L. Applying the proposed system to real-time concentration imaging, we successfully visualised unsteady concentration transport phenomena, and estimated the mass transport coefficient through the liquid-hydrogel interface. Our findings are useful for investigating the mass transport of physical, biological, and medical phenomena in permeable substrates.

## Introduction

The transport of solvent molecules through a passively permeable material is a fundamental and important dispersion phenomenon in biology, chemistry, and fluid dynamics. Brownian diffusion is governed by Fick’s law, which shows that the concentration flux is proportional to the negative spatial gradient of the concentration and diffusion coefficient of a molecule. In the case of high Péclet number, i.e. the advective velocity exceeds diffusive velocity, and the molecules are rapidly transported by the advective velocity rather than by the diffusive velocity [1, 2]. In contrast, in the case of low Péclet number, diffusion is more dominant than advection. When heat, concentration, or pH distribution is inhomogeneous, the scalar transport phenomenon is induced by the gradient, which follows Fick's law under conditions of low Péclet number [3]. Although the advective velocity is very slow under these conditions, the diffusive velocity is significantly high under micro-scale observation. Thus, the resolution in time, space, and scalar values should be improved by enhancing the equipment, camera, scanning speed of the galvanometer mirror, intensity of the laser, photo breach resistance, and quantum yield of fluorescent molecules for optical scalar measurements.

In the early days of measuring temperature distributions, thermo-chromic liquid crystals (TLC) were typically used to obtain local temperature using scattered light from the TLC with a sensitive temperature range of 1K or less[4–6]. A planar temperature measurement using TLC was performed to record the temperature distribution in a jet flow or a wake of a heated circular cylinder [4, 5]. Although there was a non-liner relationship between the incident colouring hue and temperature of the TLC, the researchers solved the problem using a calibrated fitting curve with an interpolation based on the experimental deviations [4]. Sakakibara and Adrian [6] confirmed that temperature measurements using TLC were within the narrow range of 0.7K, with an accuracy of 0.1K random error at the 95% confidence level. The LIF method yielded precise temperature measurement with ±1.5K accuracy over a measured range of at least 40K [6]. Consequently, temperature-sensitive fluorescent dyes were used for spatial temperature measurements using a laser-induced fluorescence (LIF) method instead of TLC [6–9]. The advantages of using fluorescent dyes were as follows: the fluorescent intensity is directly proportional to the exciting light intensity and the concentration of fluorescent dye; and the fluorescence intensity depends on the circumstance temperature. Moreover, noncontact-based thermometry techniques using fluorescent dyes have advantages that achieve not only simultaneous precise temperature measurement, but also no disturbance during the measurement [9]. For this reason, the LIF method for thermal measurement has been applied for a small region such as liquid flow in a microchannel [10–12].

More recently, three-dimensional concentration measurements in the microchannel have been established using laser sheet scanning driven by galvanometer mirror and multi-colour LIF methods [13, 14] combined with the micro particle image velocimetry (PIV) method [15]. The high temporal and spatially resolved digital imaging of fluorescents yields precise temperature measurements over a wide dynamic range, with LIF techniques using thermal-sensitive fluorescent dyes [16]. Three-dimensional density and velocity measurements have been performed using the 2-dimensional LIF and PIV methods with a scan rate 200 Hz of depth recording for a centimeter-scale volumetric range [17]. Recently, the LIF technique has been adapted for transport phenomena coupling with fluorescence microscopy [18] and point scanning confocal microscopy [19]. A combination of the LIF technique and microscopy has established to measure the concentration of molecules with two dimensional micro-scale spatial resolution [20]. Thus, the real-time depth concentration measurements have been achieved using the LIF technique with three-dimensional micro-scale spatial resolution [21], however, the simultaneous depth concentration measurement has not been achieved despite the fact that the measurement of transient mass transport is important for estimating physical and chemical quantities in transport phenomena.

The three-dimensional concentration distribution provides the total mass by integrating concentration into the entire measurement region, and the real-time concentration distribution yields the mass flux by differentiating the total mass by time. In fact, the mass transport coefficient is calculated by the relationship between the mass flux and the concentration difference at the interface. In transdermal drug delivery systems (TDDSs), for example, the drug permeation flux is often measured using the Franz cell [22–24], which invasively measures the permeated drug passing through the test specimen from the donor to receptor reservoirs [22]. The type of test sample, however, is very limited using the method because it is suitable for only *in vitro* measurements; this is because the reservoirs should be invasively positioned at both sides of the sample. However, measurement of the *in vivo* drug flux in skin, for example, requires measurement of the non-invasive concentration of the three-dimensional distribution. The application of two-dimensional planar or projected concentration measurements has been investigated for scanning three-dimensional concentration distributions. If the mass transport in the depth direction is measured using such scanning concentration planes, the time resolution should be sufficiently high to enable measurement of transient phenomena. To overcome this difficulty, we applied tilted observation, using microscopy, to the μPIV method used in our previous study for velocity measurements with simultaneous multi-depth positions [25]. We believe that this tilted observation would also be useful for simltaneous concentration measuring in the depth direction.

In this paper, we propose a novel approach for the measurement of mass transport in the depth direction via a real-time non-invasive method with micro-scale depth resolution using confocal microscopy with geometrically inclined observation. The technique enables measurement of the precise concentration in a hydrogel film via calibration of photo attenuation curves. In particular, the method precisely measures the time history of the permeation of molecules into transparent hydrogel. As a result, we were able to estimate the mass transport coefficient on the surface of the hydrogel film.

## Experimental setup

### Inclined confocal micro-LIF measurements

**Fig 1:**
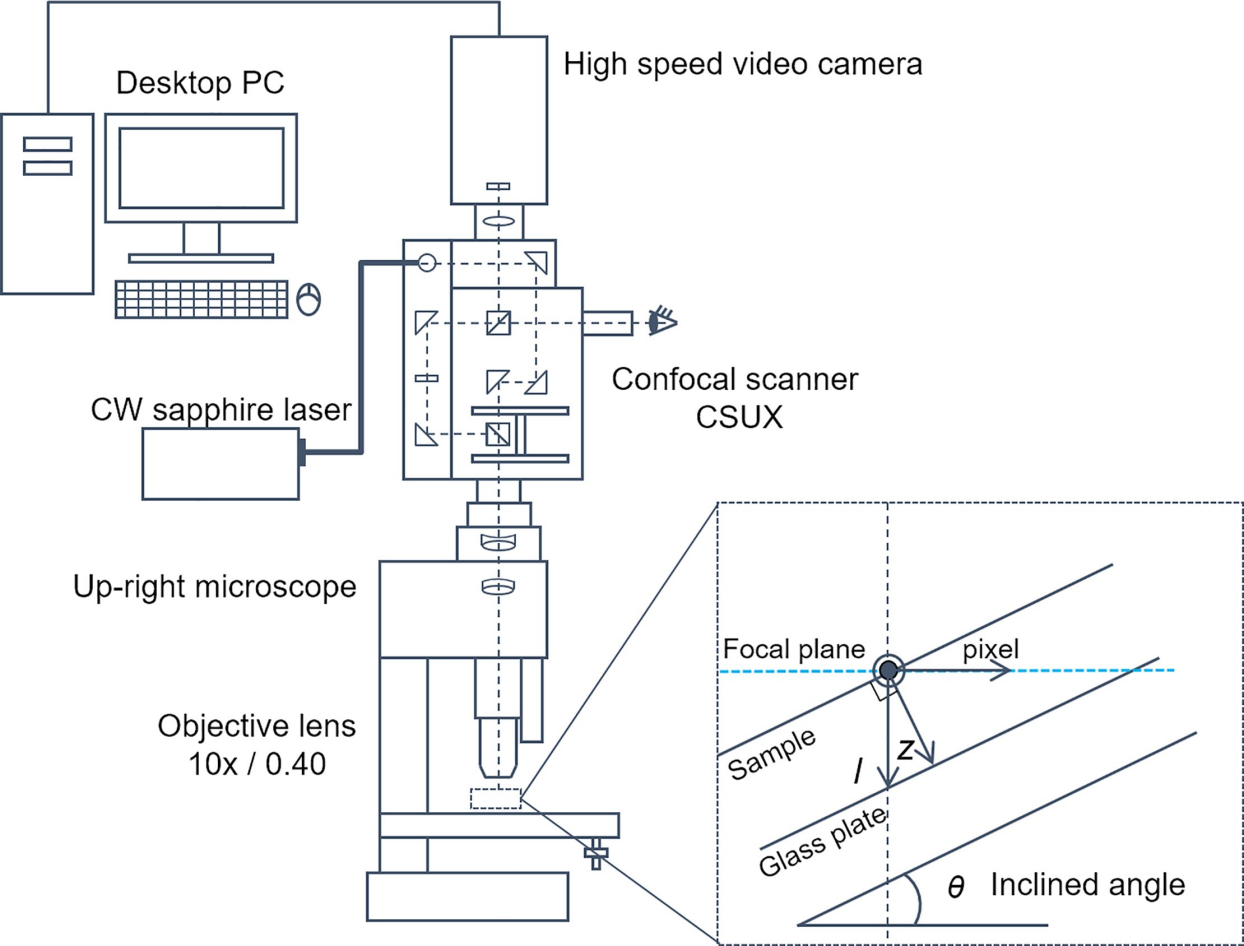
Experimental setup for depth concentration measurement using laser-induced fluorescence (LIF) with inclined microscopic observation. The close-up view shows the geometry at higher definition.

We vertically assembled a confocal spinning disc unit and a high-speed camera with high spatial resolution into a confocal micro-LIF system, as shown in Fig 1. The confocal spinning disc microscope comprised an optical microscope (BX51; Olympus, Japan) with a 10× (NA = 0.4, WD = 1.0 mm) objective lens (Uplsapo Fl; Olympus), a confocal scanning unit (CSUX, ~10,000 rpm; Yokogawa, Japan) and a CW sapphire laser (488nm, ~100 mW; Coherent, Japan). A high-speed video camera (SA3; Photoron, Japan) was installed at the external port of the CSUX. Viewing positions were adjusted using a piezo actuator (RT3D; Yokogawa) between the microscope and objective lens. The high-speed video camera and piezo-controller were controlled by Koncerto software (ver. 2.0; Seika Corp., Japan). A flat glass plate (micro-slide glass S1111; Matsunami Glass Ind., Ltd., Japan) was mounted at an inclined angle *θ* of 25° for inclined observation, which geometrically enables a depth field measurement as being -240 μm depth in the transparent sample [25].

The spinning disc equipped with micro-lenses and the pinholes of the CSUX scanner unit formed an optical thin focal plane through array of pinholes (50 μm in diameter), which shut out scattered light paths from defocus points. The Nipkow spinning disc could rotate at up to 10,000 rpm and was installed with 20,000 pairs of micro-lenses. An optical sliced picture was captured by approximately 2,000 scanning beams at a 0.5 ms interval. The spatial resolutions of the optical slice, i.e. its lateral, axial and thickness resolutions, were calculated from the equations given by Park et al. [26]. Following their methods, the spatial resolution of our equipment was calculated from the illuminating laser wavelength, emission wavelengths of the fluorescence particles, numerical aperture of the objective lens, and refractive index [27–29]. The optical section thickness (OST), i.e. the full-width half-maximum of the intensity profile in the z-direction, was 24.5 μm according to the optical theoretical equation [26], which were in good agreement with a measured OST as being 29.2 μm [30]. The optical sheet illuminated the sample and excited the fluorescent material, and the resulting fluorescence intensity was captured on an image sensor through a band pass filter (525 ± 25 nm). The filtered images were transmitted to a high-speed video camera at a resolution of 580 × 370 pixels (576 μm × 368 μm) in the field of view (FOV), yielding a spatial resolution of 0.99 μm/pixel for the LIF-images. The background light brightness in the FOV was corrected as to be flattened using a standard shading correction technique [31] in advance; hence, the spherical aberration of the objective lens could be neglected. The video camera operated at a maximum of 60 frames per second with 12-bit mono colour. The frame rate was synchronised with the scanning rate at 5,000 rpm in the scanning speed of CSUX. The high-speed video camera was limited to 60 frames per second by the low intensity of the light emitted from the fluorescence material, which depended on the laser power within a range of 10–100 mW.

### Materials

Uranine solution (fluorescein sodium salt, molecular weight: 376.27; TCI, Japan) was used as the fluorescence material, and served as the concentration indicator. Since uranine has a maximum absorption peak at 490 nm and maximum fluorescence at 513 nm with pH 7.0–7.5, we used a coloured filter in the 525 ± 25 nm band at the output of the confocal scanner. Phosphate-buffered saline (PBS) solutions containing fluorescent dye were prepared in advance with 5, 10, 25, 50, 75, and 100 μmol/L uranine. Several concentrations of uranine solution were diluted from 100±2 μmol/L uranine by PBS, which was prepared using an electrical scale with 0.2 mg in minimal resolution and a standard glass measuring cylinder with 0.8% error. Three wt% agarose (Agarose gel ME; Wako, Japan) was melted in PBS solution (pH7.4; Thermo Fisher Scientific, USA) containing uranine at 100°C for 30 min on a heating magnet stirrer. The agarose hydrogels for calibration experiments were moulded in between glass slips as hydrogel sheets 170 μm in thickness, at room temperature (25°C) for 30 min.

We prepared single and double hydrogel layers for our experiments. The double-layered hydrogels were formed by vertically stacking two different hydrogel layers, containing 25 or 50 μmol/L uranine. Double hydrogel layers with each 170 μm in thickness were separated using thin non-permeable plastic film 10 μm in thickness to prevent transport of the uranine through the hydrogel interfaces.

**Fig 2:**
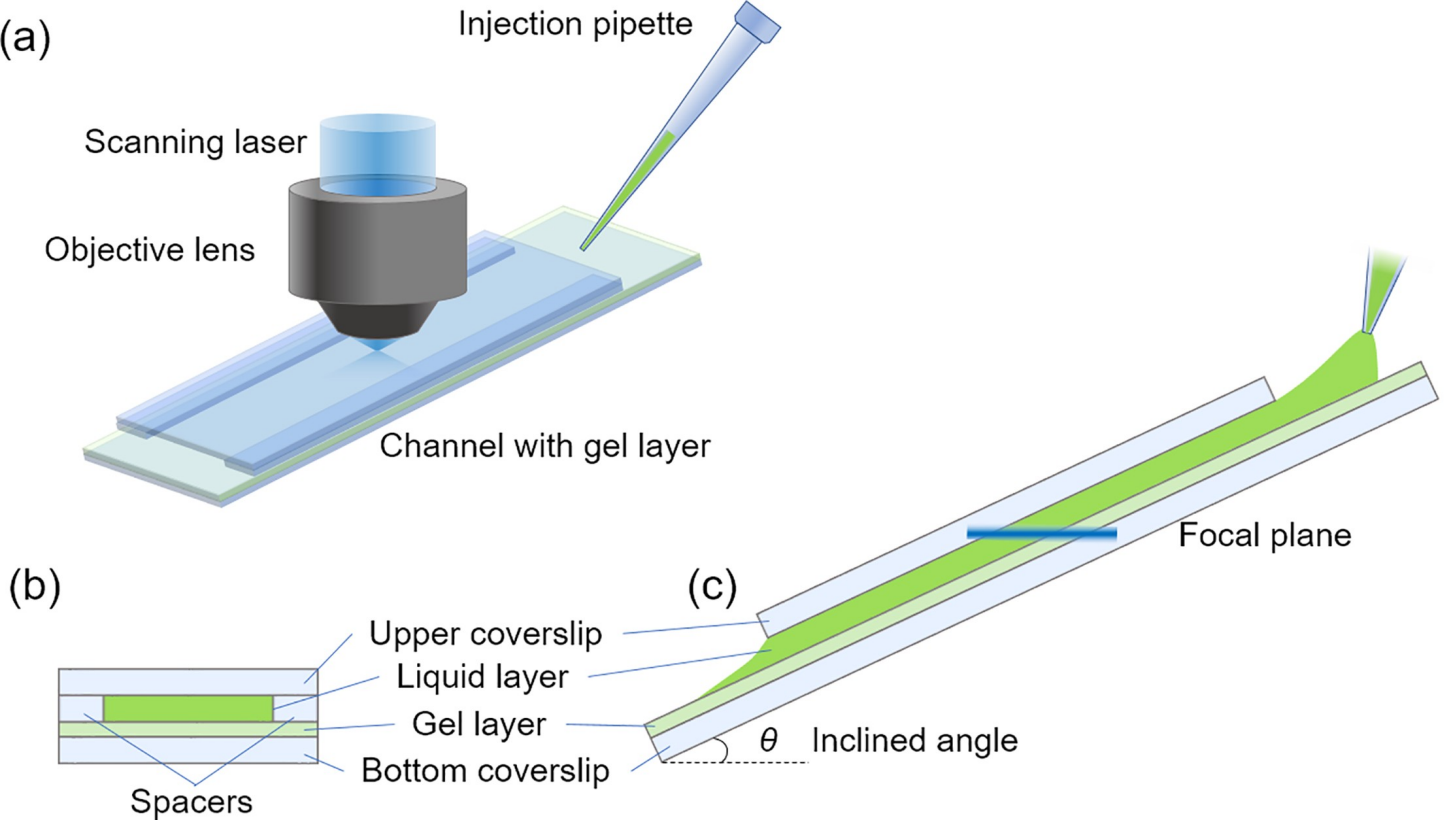
Schematics of transient molecular transport measurement using inclined microscopic observation. (a) Bird view of experimental setup, (b) Cross-sectional view of channel with gel layer, (c) Side view of a channel with observed focal plane.

To observe the transient transport phenomenon thorough the liquid-hydrogel interface, we made a channel for liquid flow on the hydrogel using a cover slip with spacers as shown in Fig 2. The upper liquid layer had a thickness of 170 μm containing an uranine concentration of 100 μmol/L. To maintain a concentration of 100 μmol/L in the upper layer, we repeatedly injected the uranine solution with a syringe. The lower hydrogel layer had a thickness of 140 μm and an initial uranine concentration of 20 μmol/L.

### Theory for three-dimensional concentration measurement

The fluorescence intensity of a fluorescent molecule with absorption of emitting light shows linearity in its concentration, and is given by

$$I=I_{e}\varepsilon \varphi C,$$ (1)

where *I* is the fluorescence intensity, *I*_*e*_ is the excitation light intensity, *ε* is the extinction coefficient, *φ* is the quantum yield and *C* is the concentration of molecule. The fluorescence light intensity at several depth positions in the aquatic permeable wall, *I*(z), is attenuated according to the light pass length, *l* = z/cos*θ* (cf. Fig 1). We ignored the attenuation of the light signal in the air. The attenuation of the excitation light intensity through the hydrogel is defined by the Lambert-Beer law [32, 33],

$$I_{e}=I_{0}exp(-\Omega (C)l)$$ (2)

where *I*_*0*_ is the incident light intensity, and Ω(*C*) is the attenuation function, which is associated with *ε* and *C*. Thus, the fluorescence intensity at several depth positions can be rewritten as follows:

$$I(z)=\varepsilon \varphi C(z)I_{0}exp\left(-\frac{1}{cos\theta }\int_{0}^{z}\Omega (C)dz\right).$$ (3)

The concentration at several depth positions, *C*(*z*), can be derived from the following equation:

$$C(z)=\frac{I(z)}{\varepsilon \varphi I_{0}}exp\left(\frac{1}{cos\theta }\int_{0}^{z}\Omega (C)dz\right).$$ (4)

The concentration at the surface (*z* = 0) with no attenuation, *C*(0), is given by:

$$C(0)=\frac{I(0)}{\varepsilon \varphi I_{0}}.$$ (5)

Here, *I*(0) is the fluorescence intensity at the surface. By measuring *I*(0) under a given *C*(0) condition, we can derive *εφI*_0_ from Eq (5). Then, knowing the attenuation function Ω(*C*), we can derive *C*(*z*) from Eq (4) by measuring *I*(*z*).

## Results

### Fluorescence intensities in the hydrogel containing homogenous fluorescence material

**Fig 3:**
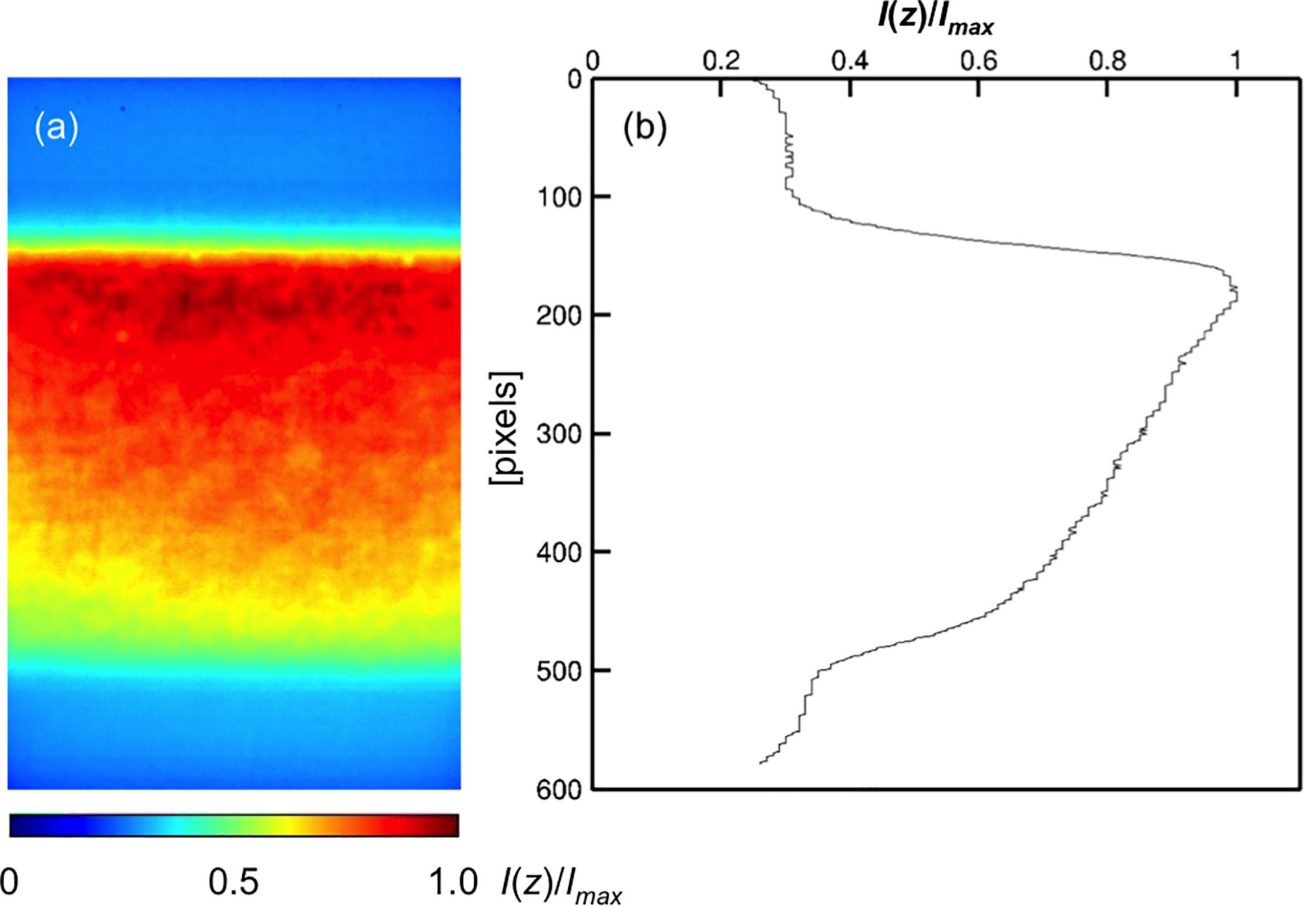
Fluorescence intensity at several depth positions measured using our method. (a) Fluorescence intensity distribution in the hydrogel containing a homogeneous concentration of 100 μmol/L uranine. The thickness of the hydrogel was 170 μm. The coloured contours show the fluorescence intensities normalised by the maximum intensity. (b) The averaged fluorescence intensity in the hydrogel.

**Fig 4:**
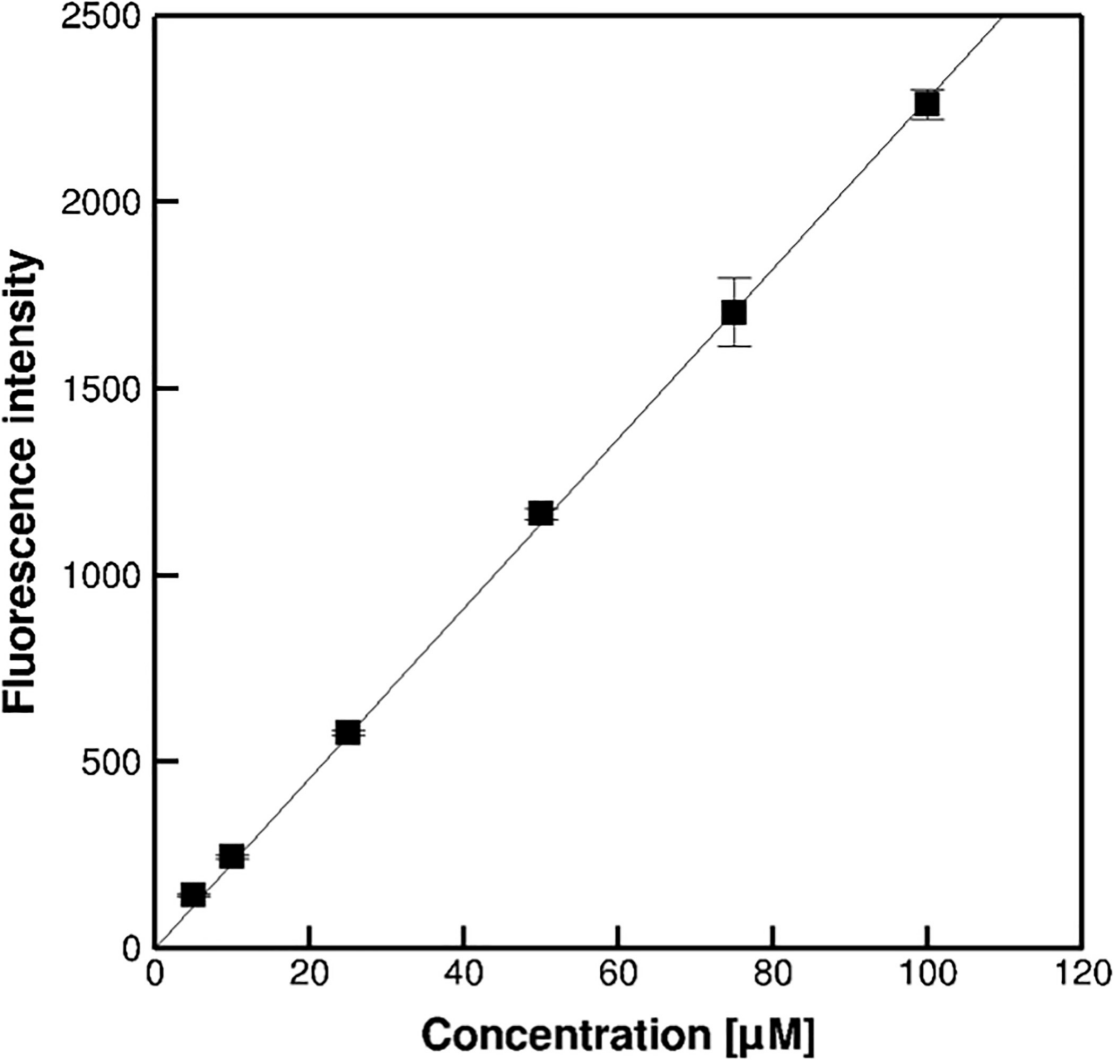
Fluorescence intensities at the surface of the hydrogel with different concentrations of uranine. The error bars show standard deviations (N = 10).

The fluorescence intensities in the depth direction were obtained in the agarose gel with 100 μM uranine, as shown in Fig 3(A). The colour bar shows the scale of normalised fluorescence intensities, *I*(*z*)/*I*_0_. The fluorescence intensities gradually attenuated from the surface to the bottom, as shown in Fig 3(B), although the concentration of uranine was homogeneous. The maximum intensity appeared near the top surface of the hydrogel. The fluorescence intensities were high, in the range in FOV with 110–500 μm, i.e. corresponding to a length of 390 μm. By multiplying sin*θ* with 390 μm, we obtained a thickness of 165 μm, which was similar to the hydrogel thickness of approximately 170 μm. The upper and lower interfaces had blunt edges in the fluorescence intensities, which was caused by the Gaussian intensity distribution with depth of the OST [30, 34]. In our tilted observation condition, the Gaussian intensity distribution may be influenced abnormally by optical aberrations, which cause aberrance light path by refractive index mismatch from objective lens to focal plane. Namely, the fluorescence intensity was produced by a convolution between the intensity distribution of the OST and the spatial distribution of the fluorescence molecules. Furthermore, the measured fluorescence intensities were obtained as the spatially averaged values in the OST. The fluorescence intensities in the hydrogel were exponentially attenuated, as described in detail below. Fig 4 shows the relationship between the fluorescence intensity *I*(0) and the uranine concentration *C*(0) at the surface. The linear approximation was in close agreement with our results, confirming the validity of the proportionality assumed in Eq (5). We obtained the coefficient*εφI*_*0*_ in Eq (5) by measuring the slope in Fig 4.

### Attenuation of fluorescence intensity in the depth positions in the hydrogel

**Fig 5:**
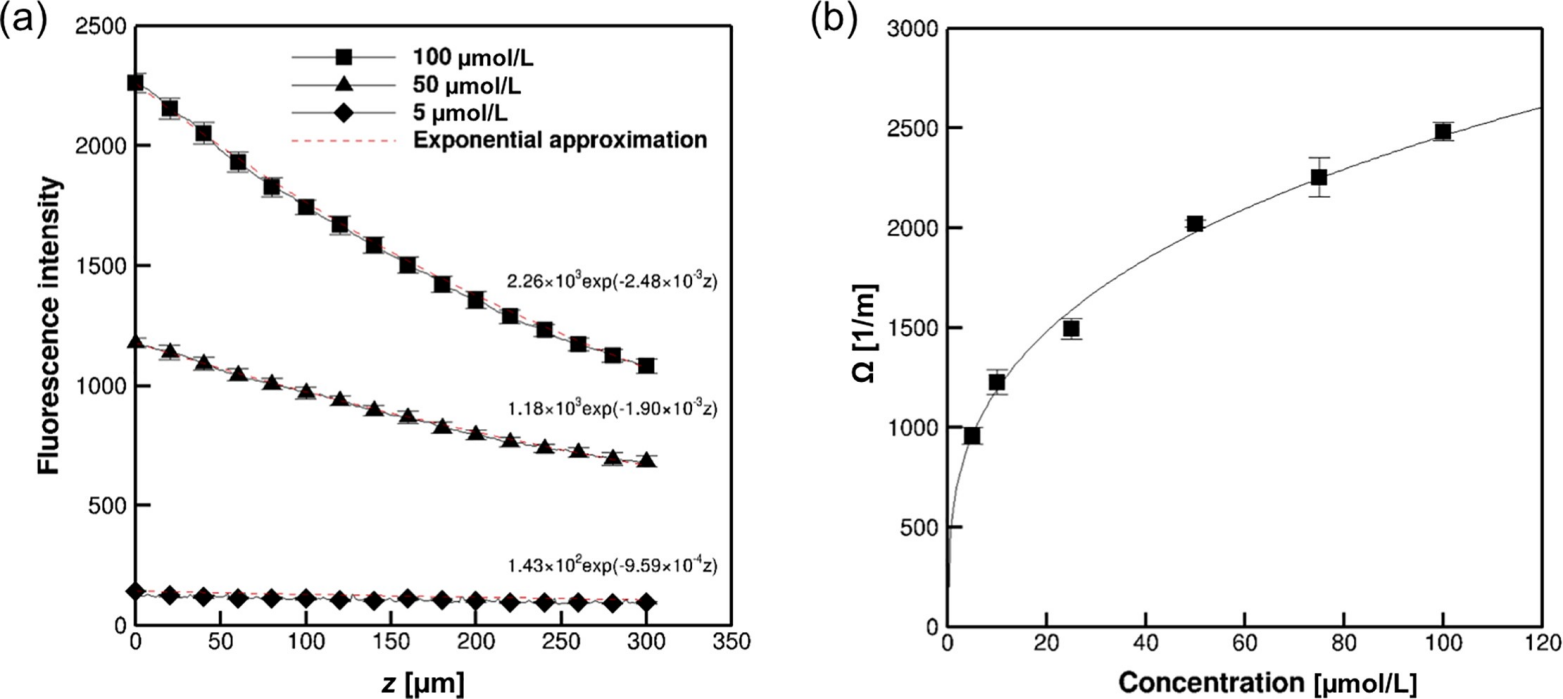
Attenuation of fluorescence intensity in the hydrogel containing different homogeneous concentrations of uranine. (a) Fluorescence intensity profiles with depth position at concentrations of 5, 50, and 100 μmol/L. The broken lines show the exponential fittings. (b) Change in attenuation function Ω with uranine concentration, *C*.

Fig 5(A) shows the attenuation of the fluorescence intensity in the hydrogel at several depth positions for uranine concentrations of 5, 50, and 100 μmol/L. The depth interval for all concentrations was 1.1 μm/pixel. The error bars show the standard deviations. The attenuations increased with increasing concentration. To obtain Ω(*C*), we fitted the attenuation curves at concentrations of 5, 10, 25, 50, 75, and 100 μmol/L using exponential functions, shown as broken lines in Fig 5(A). The attenuation function at the surface Ω(*C*) is shown in Fig 5(B), which displays a logarithmic increase with increasing uranine concentration. Henceforth, we were able to derive the concentration distribution in the hydrogel using Eq (4) by adopting *C*(0) and integrating the Ω(*C*) value from the surface.

### Molecular concentration depth measurement in the hydrogel

**Fig 6:**
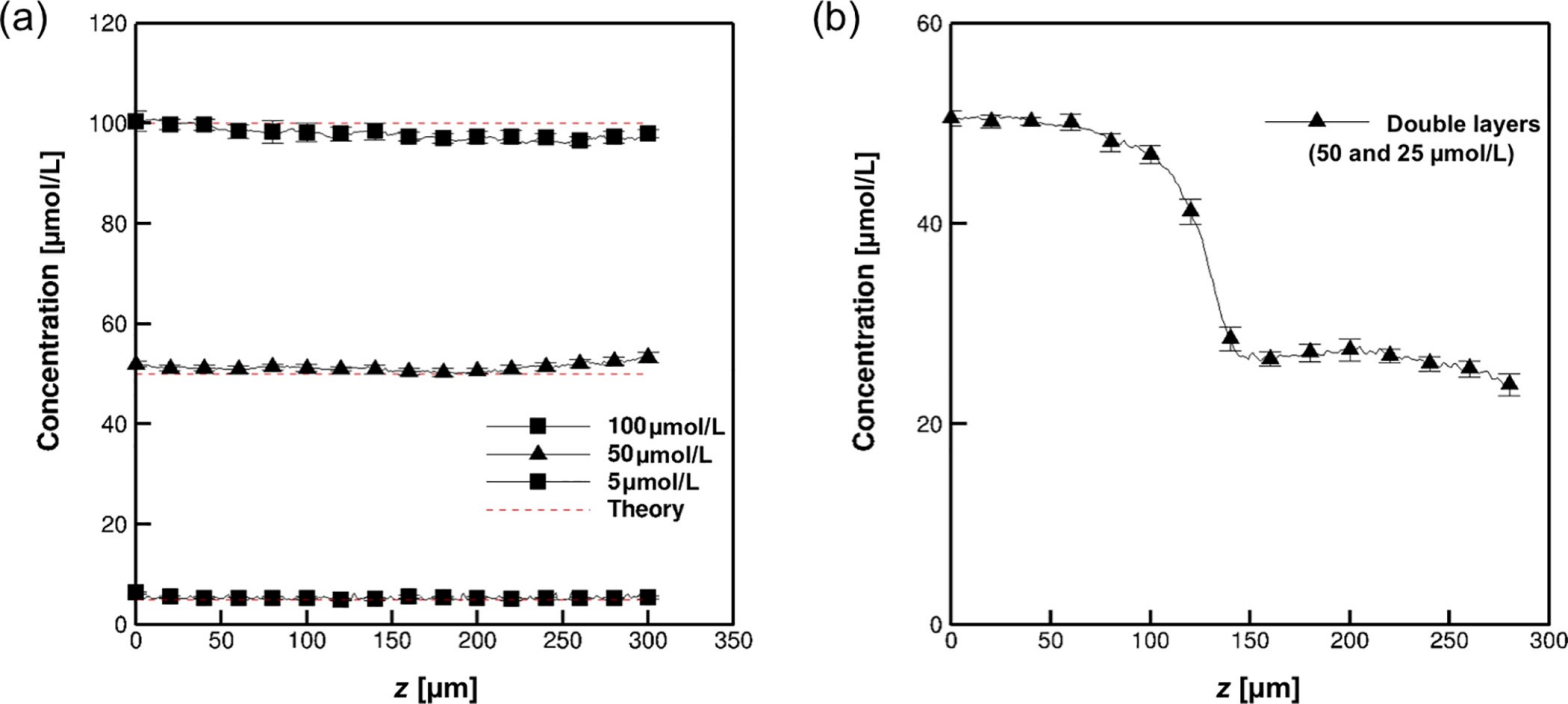
Estimated depth concentration of uranine in the hydrogel using one-shot measurements. (a) Estimated concentration in single hydrogel layers containing 5, 50, and 100 μmol/L uranine. Broken lines show the theoretical values. (b) Estimated concentration in double hydrogel layers at different uranine concentrations (50 and 25 μmol/L). A non-permeated transparent layer 10 μm in length was placed at the interface to prevent mass transport through the interface.

Fig 6(A) shows the estimated concentration of uranine at several depths in the hydrogel containing homogeneous uranine concentrations of 5, 50, and 100 μmol/L. The broken lines show the theoretical concentrations. The estimated concentrations in the hydrogel were in close agreement with theoretical values. The deviations in the measured concentrations were calculated from the following equation:

$$error\%=\left|\frac{Estimated\;concentration-Actual\;concentration}{Actual\;concentration}\right|\times 100.$$ (6)

The deviations were large near the edge of the hydrogel, with a maximum error of 6.8%. The accuracy of the concentration measurements using our system was 1.3 nmol/L, which was calculated from the dynamic range of intensity as being 12-bit (4,096 grey level) with 6.8% experimental error for a concentration of 5 μmol/L.

Next, we examined the concentration measurements using double hydrogel layers, with an upper layer concentration of 50 μmol/L and a lower layer concentration of 25 μmol/L. Fig 6(B) shows the estimated concentration distribution in the double hydrogel layers. We can see that the estimated concentrations are approximately 50 and 25 μmol/L in the upper and lower layers, respectively. At the interface of the two layers, the fluorescence intensities displayed a blunt distribution, which was again caused by a Gaussian intensity distribution with depth of the OST [30].

### Real-time visualisation of molecular permeation in the depth direction

**Fig 7:**
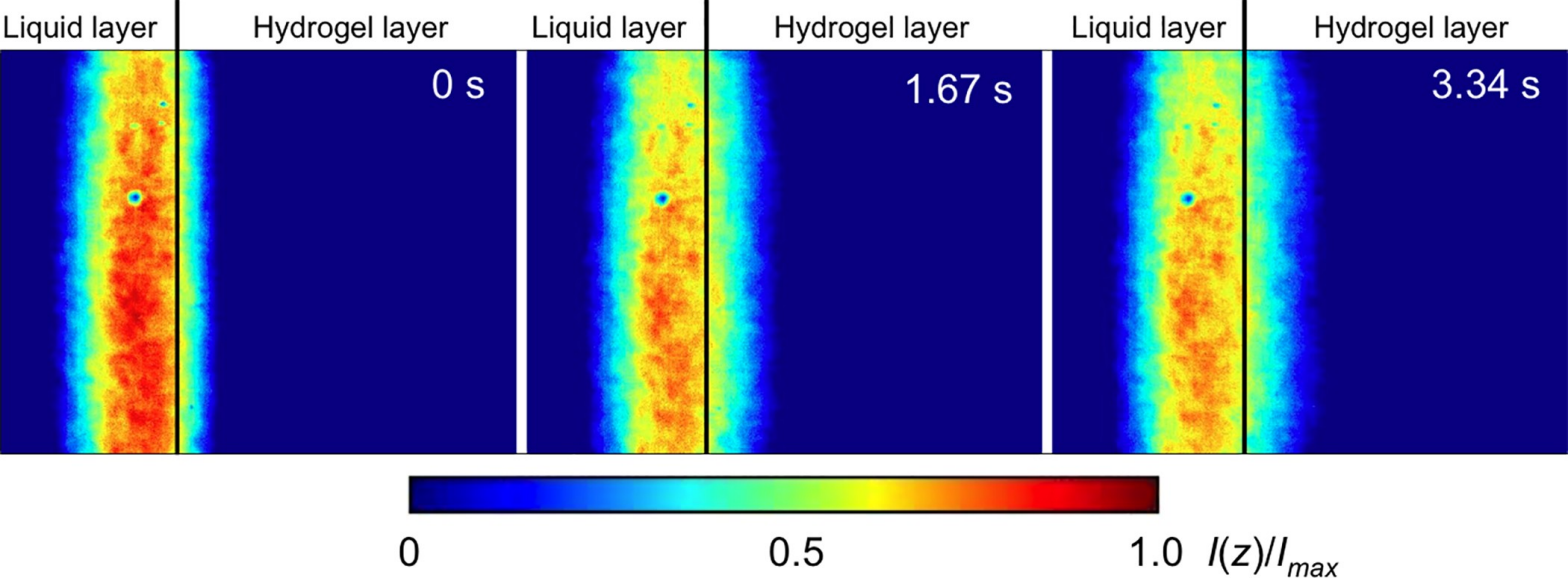
Transient permeation of uranine from the upper liquid layer to the lower hydrogel layer at a time resolution of 16.7 ms. Each set of 100 steps of time-lapse images is sequentially shown as a time step of 1.67 s. The coloured bar shows the normalised fluorescence intensities.

The transient permeation of uranine from the liquid layer to the hydrogel layer was visualised at 60 frames per second, and with a laser intensity of 25 mW using inclined confocal microscopy. Fig 7 shows the time-lapse images of the transport of fluorescent intensities at 16.7 ms intervals. The coloured bar shows the normalised fluorescence intensity of uranine. To maintain a constant uranine concentration in the upper liquid layer, we repeatedly injected 100 μmol/L uranine solution during the experiment. The hydrogel initially contained 20 μmol/L uranine to estimate the surface of the hydrogel layer. The measurement position was set in advance before introducing the flow of uranine solution.

**Fig 8:**
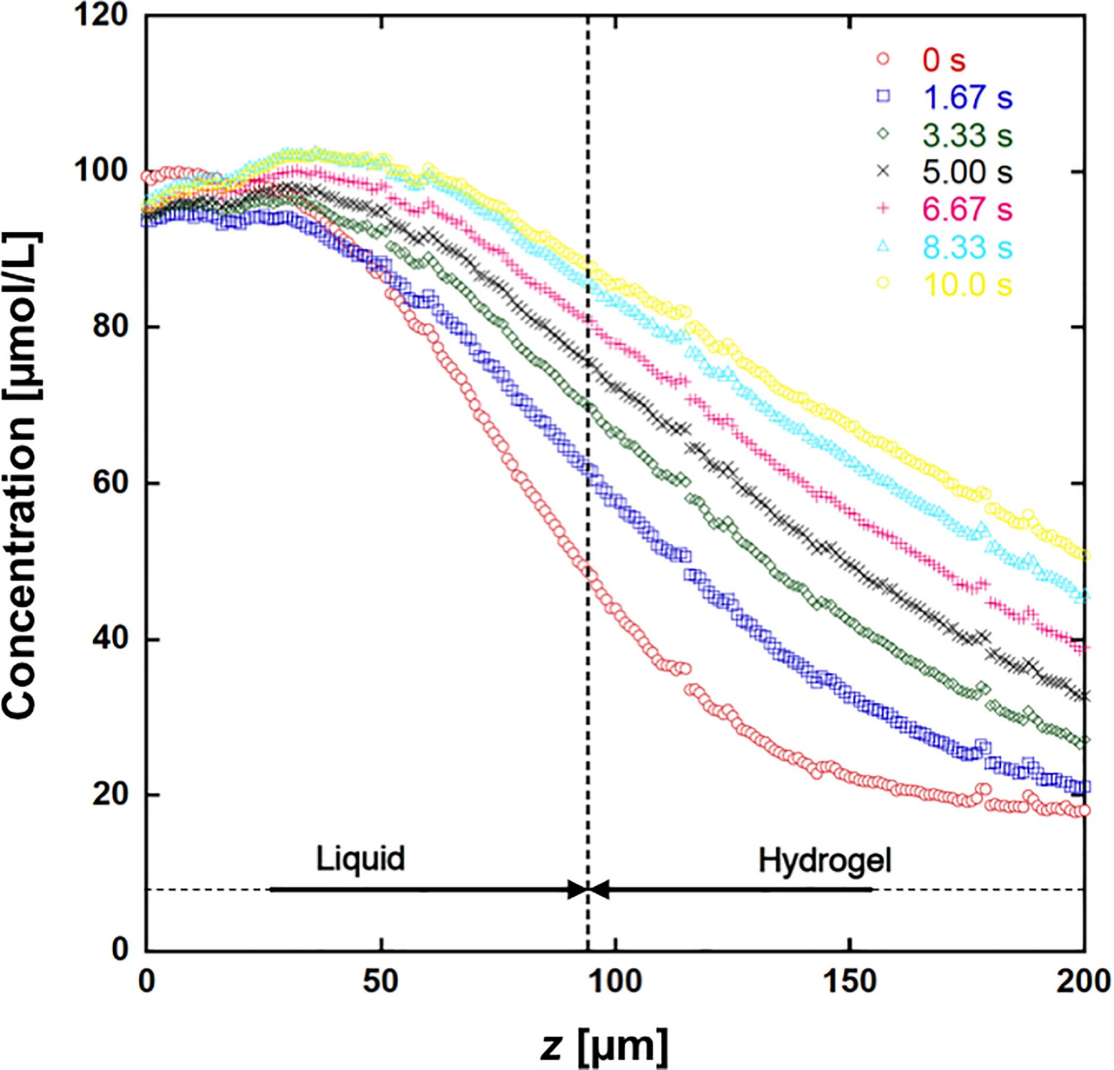
Temporal change in the concentration distributions in the liquid and hydrogel layers. The liquid layer contained 100 μmol/L uranine, while the hydrogel layer contained 20 μmol/L uranine. The broken line shows the interface of liquid and hydrogel, in which concentrations were initially 100 and 20 μmol/L, respectively.

The uranine solution was injected at *t* = 0. Then, the fluorescence intensities in the hydrogel were gradually increased by mass transport at the interface between the liquid and hydrogel layers (see S1 Movie). Fig 8 shows the time history of the concentration distribution in the double layers. The estimated concentrations around 100 μmol/L had some fluctuations, which were within the maximum measurement error of 6.8%. This measurement error would include at least thermal error, which is caused by the thermal sensitivity of fluorescence of uranine with 2.0% error for 1K difference [35]. The upper region in the liquid layer maintained a stable concentration of 100 μmol/L, because we maintained a flow of 100 μmol/L uranine solution during the experiment. At *t* = 1.67 s, the uranine had not reached the bottom of the hydrogel layer. Subsequently, uranine gradually accumulated in the hydrogel layer, and its concentration increased with time.

## Discussion

Attenuation of fluorescent intensity was caused by the extinction of excitation radiation. In the dilute limit condition of fluorescein concentration (~20 μmol/L), the attenuation should increase linearly with increasing the concentration of molecules [36]. This is because the individual molecules obstruct the light path and the light intensity is decreased as the concentration is increased. In the case of dense fluorescein concentration, on the other hands, the linearity would be lost, because the fluorescent molecules may overlap in each light path. This might be the reason why the attenuation showed non-linearity and slope was decreased as the concentration was increased as in Fig 5(B).

Mass transport from the liquid layer to the hydrogel layer can be expressed using the mass transport coefficient, *κ*, as:

$$q=\kappa (C_{b}-C_{i}).$$ (7)

where *q* is the mass flux with units of mol/(s m^2^), *κ* is the mass transport coefficient with units of m/s, *C*_*b*_ is the bulk concentration, and *C*_*i*_ is the concentration at the interface. The mass transport coefficient *κ* is a key physical quantity for calculating the mass transport through a surface. Thus, we calculated the value of *κ* from Eq (7) by inserting the mass flux, *q*, and the concentration at the interface, *C*_*i*_, both of which were estimated from our measurements.

**Fig 9:**
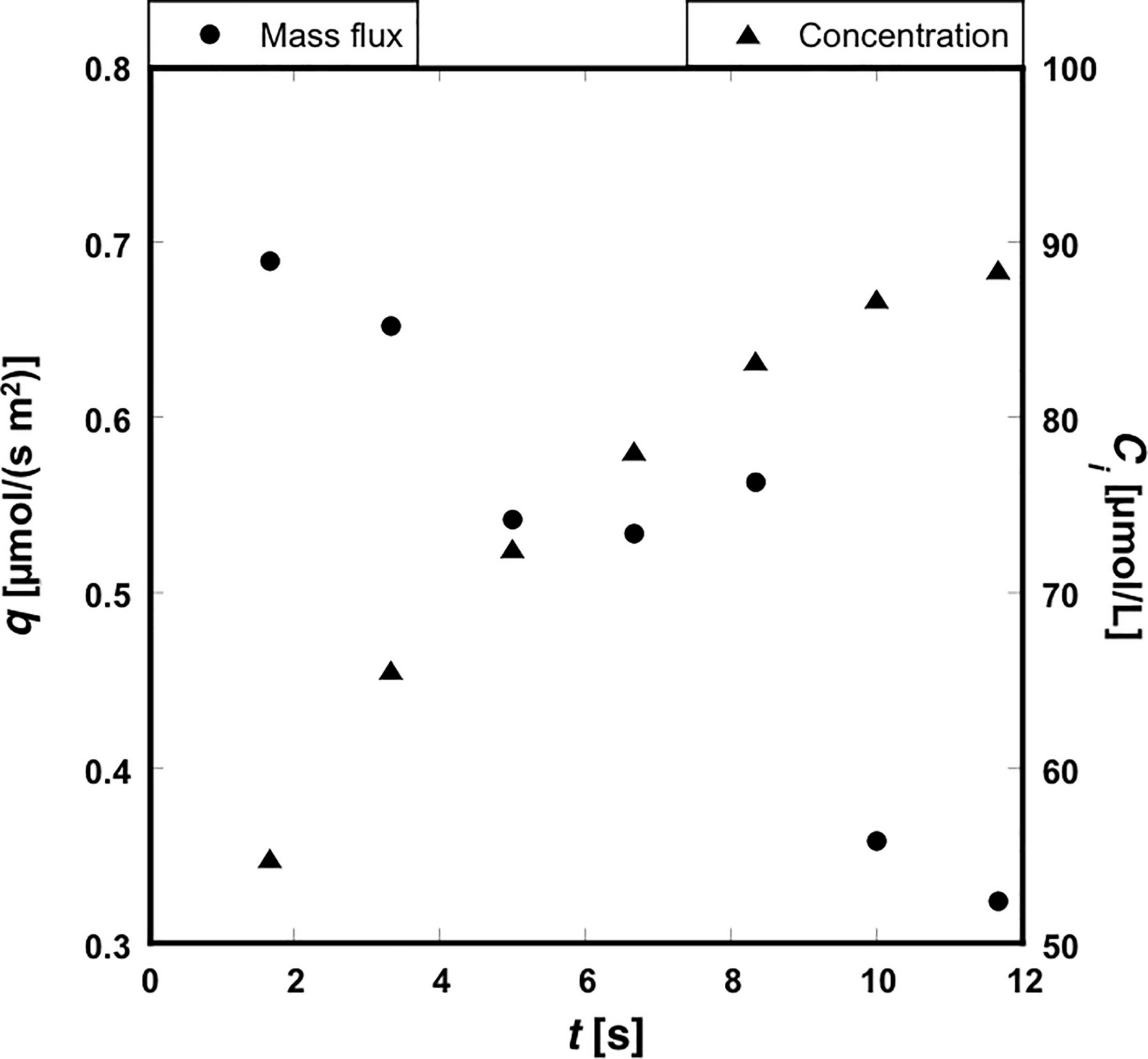
Temporal change in mass flux, *q*, and concentration at the interface, *C*_*i*_.

Using the measured concentration in the hydrogel, we estimated the mass flux through the interface, *q*, by differentiating the mass by time. The mass of uranine was calculated by integrating the uranine concentration in the entire hydrogel layer. The concentration distribution outside the observed region was estimated as follows: the transport of uranine inside the gel layer was assumed to be a one-dimensional diffusion process, given that the thickness of the gel layer was significantly smaller than the channel width and length. Because uranine could not penetrate the bottom glass plate, the boundary condition on the bottom plate was a ‘no-flux’ condition. The analytical solution for the one-dimensional diffusion process with a no-flux boundary condition is available [3]. We employed the analytical equation to extrapolate the concentration distribution outside the observed region, using the least squares method under the condition of the analytical curve passing through the experimental value at *x* = 200 μm. The mass flux *q* was then calculated by dividing the difference in total mass by the time duration. The change in *q* with time is shown in Fig 9, which shows that *q* decreased monotonically with time. Since the injected flow on the upper layer fluctuated, and was not fully developed in the present study, the mass flux also fluctuated as shown in Fig 9.

**Fig 10:**
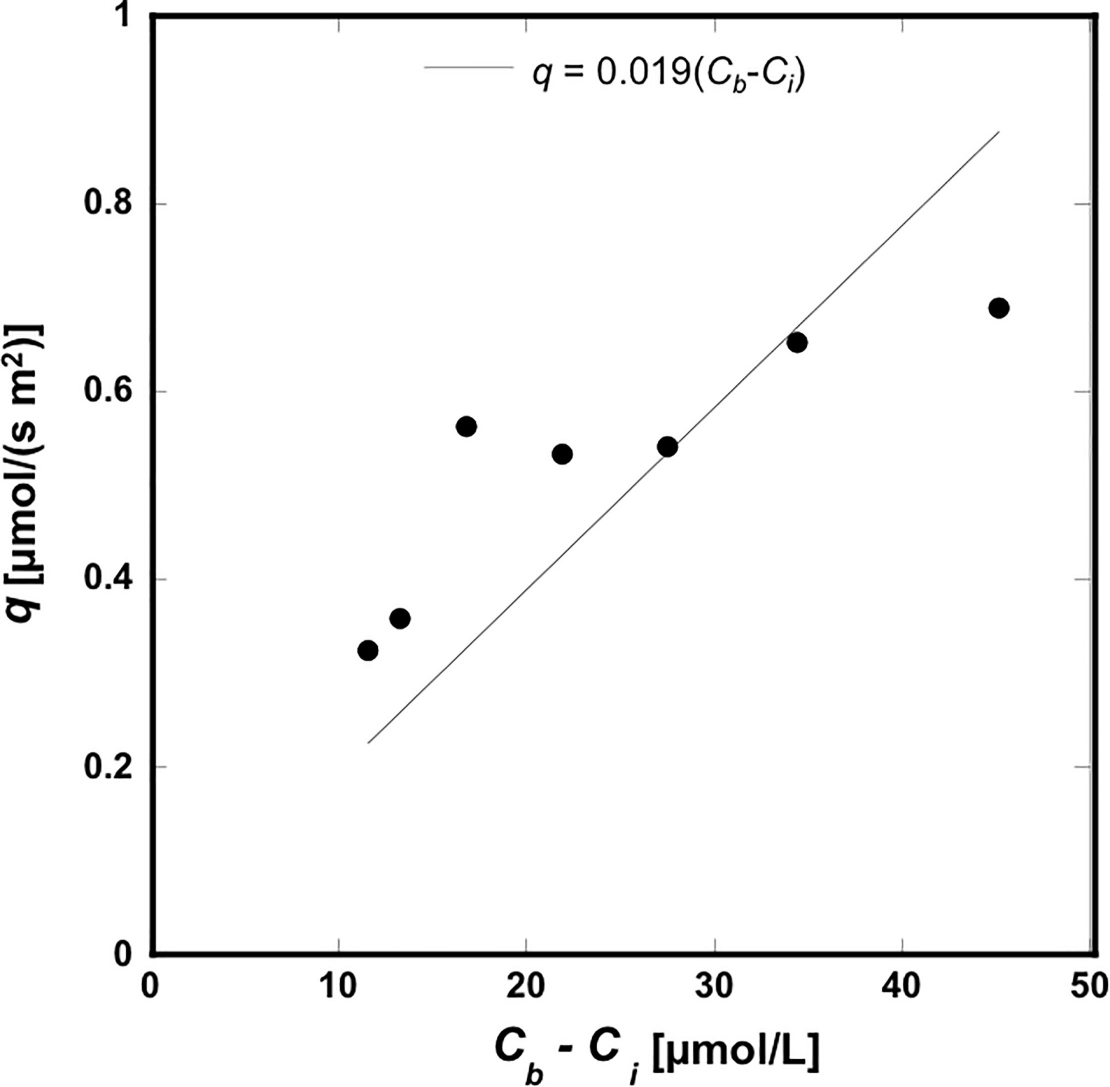
Correlation between the mass flux, *q*, and the concentration difference, *C*_*b*_−*C*_*i*_. A fitted line passing the origin was plotted using the least squares method.

*C*_*i*_ in Eq (7) could be approximated as the measured concentration at the interface. The results are also shown in Fig 8, which shows that the concentration at the interface increased gradually with time. *C*_*b*_ in Eq (7) could be approximated as 100 μmol/L, given that 100 μmol/L uranine solution was continuously injected into the upper liquid layer. Once the values of *q*, *C*_*i*_, and *C*_*b*_ had been obtained, we could calculate *κ* from Eq (7). The correlation between *q* and (*C*_*i*_−*C*_*b*_) is plotted in Fig 10. By fitting a line passing through the origin by the method of least squares, we estimated *κ* as 19.4 μm/s.

To obtain the value of *κ*, it is necessary to measure the mass flux, *q*, which requires accurate measurement of the temporal change in the concentration distribution in the depth direction. Our proposed method offers high resolution in both time and depth, appropriate for measuring the value of *κ*. Furthermore, our method can non-invasively observe transient mass transport from the top of the sample through the transparent layer. We believe that our proposed method would be useful in measuring the mass transport into permeable films in various physical, biological, and medical applications.

## Conclusion

We accurately measured the depth concentration distribution in fluorescent material in transparent hydrogel using a newly developed inclined observation technique with confocal microscopy. Estimation of the concentration in the hydrogel containing fluorescent material was performed using a combination of the attenuation theory of the Lambert-Beer law and the conventional laser-induced fluorescence method. The accuracy of the concentration measurements was 1.3 nmol/L with a depth interval of 0.99 μm. The transient passive transport by the concentration gradient was measured non-invasively in real-time using our system. Our method also yielded the mass flux coefficient of uranine through the water-hydrogel interface. Our findings are useful for determining the mass transport in permeable substrates in physical, biological, and medical phenomena.

## Supporting information

S1 Movie Transient permeation of uranine from the upper liquid layer to the lower hydrogel layer at a time resolution of 16.7 ms. The normalised fluorescence intensities were indicated in pseudo colours as seen in Fig 7.
(AVI)
